# Conversion of Similar Xenochemicals to Dissimilar Products: Exploiting Competing Reactions in Whole-Cell Catalysis

**DOI:** 10.3390/molecules28135157

**Published:** 2023-07-01

**Authors:** Francesca Sannelli, Nikoline Corell Sindahl, Stefan S. Warthegau, Pernille Rose Jensen, Sebastian Meier

**Affiliations:** 1 Department of Chemistry, Technical University of Denmark, Kemitorvet, Bygning 207, 2800 Kongens Lyngby, Denmark; fransa@kemi.dtu.dk (F.S.);; 2 Department of Health Technology, Technical University of Denmark, Elektrovej 349, 2800 Kongens Lyngby, Denmark

**Keywords:** carboligation, D-DNP NMR, in-cell NMR, pyruvate decarboxylase, substrate mixtures, sustainable chemistry, whole-cell catalysis, yeast catalysis

## Abstract

Many enzymes have latent activities that can be used in the conversion of non-natural reactants for novel organic conversions. A classic example is the conversion of benzaldehyde to a phenylacetyl carbinol, a precursor for ephedrine manufacture. It is often tacitly assumed that purified enzymes are more promising catalysts than whole cells, despite the lower cost and easier maintenance of the latter. Competing substrates inside the cell have been known to elicit currently hard-to-predict selectivities that are not easily measured inside the living cell. We employ NMR spectroscopic assays to rationally combine isomers for selective reactions in commercial *S. cerevisiae*. This approach uses internal competition between alternative pathways of aldehyde clearance in yeast, leading to altered selectivities compared to catalysis with the purified enzyme. In this manner, 4-fluorobenzyl alcohol and 2-fluorophenylacetyl carbinol can be formed with selectivities in the order of 90%. Modification of the cellular redox state can be used to tune product composition further. Hyperpolarized NMR shows that the cellular reaction and pathway usage are affected by the xenochemical. Overall, we find that the rational construction of ternary or more complex substrate mixtures can be used for in-cell NMR spectroscopy to optimize the upgrading of similar xenochemicals to dissimilar products with cheap whole-cell catalysts.

## 1. Introduction

The progress of society hinges on the sustainable production of diverse organic chemicals for materials and energy applications. Sustainable means to produce chemicals are much needed, as the vast majority (~95%) of organic chemicals in the chemical industry are still sourced from fossil resources [1,2]. Sustainable chemistry calls for approaches that yield high product quality through highly (stereo-)selective reactions using cheap catalysts and benign solvents, to provide beneficial environmental and economic metrics in production [3]. Many of these requirements are met by biocatalysis using enzymes or whole microbial cells in water, while chemocatalysis remains predominantly performed in organic solvents. Currently, chemocatalysis remains the more promising approach to large-scale production, while biocatalysis is promising in the production of high-value chemicals on a smaller scale [4,5,6]. In addition, innovative approaches at the interface of biological and chemical catalysis have been sought [5,6,7,8,9]. For instance, integrated approaches within water or biphasic solvents systems continue to be developed. Similarly, the use of chemocatalysis in water and the use of biological catalysts—both enzymes and microorganisms—in organic synthesis have rapidly gained interest [7,10,11,12]. The future uses of chemocatalysis and biocatalysis will hence depend on the target chemical and on future developments in both fields and at their interface.

The disadvantages of biocatalysis have been considered to include the often-limited capacity of enzymes to form C-C bonds and tolerate diverse reactants due to the high substrate specificity of many enzymes. By contrast, an increasingly appreciated opportunity in the biocatalytic production of organic chemicals is the incorporation of heteroatoms beyond oxygen and nitrogen into chemicals. Hence, developments to evaluate the tolerance of enzymes to heteroatom-containing organic chemicals such as aryl halides and their use as precursors in bioproduction have gained traction recently [13,14,15].

**Scheme 1:**
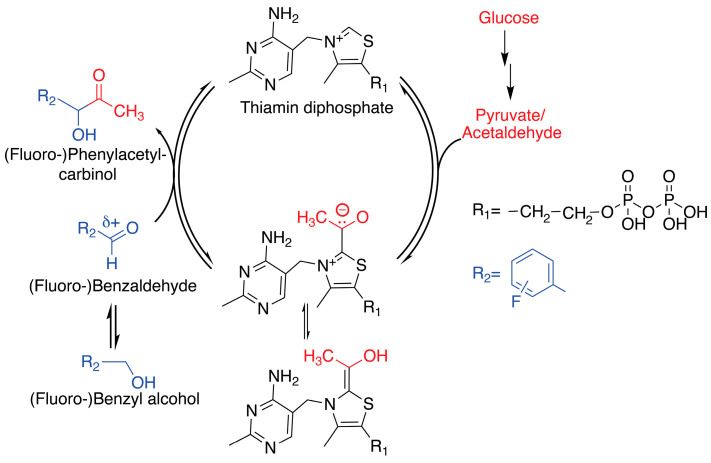
Schematic reaction cycle in the thiamine diphosphate cofactor-dependent C-C bond formation based on an umpolung. The specific example shows the reaction between glucose and benzaldehyde as well as substituted benzaldehydes in whole cells.

The ability of biocatalysts to use non-natural reactants hinges on enzyme catalytic polyreactivity (promiscuity). Thus, an increasing number of enzymes are known to be less specific to the natural substrate than had been anticipated [16,17,18]. Such promiscuity to various substrates notably includes enzymes that can be used for carbon bond extensions, many of which depend on cofactors [19] such as the thiamine diphosphate cofactor that functions in an umpolung reaction, rendering substrate carbonyl groups nucleophilic (Scheme 1). Such reactions result in α-hydroxy ketones, which are a common structural motif in high-value fine chemicals and pharmaceuticals [20] that have been obtained using both intact cells and purified thiamine diphosphate-containing enzymes. While purified enzymes promise higher reaction yields due to the absence of the detrimental competing reactions occurring inside intact cells, cells can be many orders of magnitude cheaper and more stable. In addition, intact cells may allow the use of a cheaper substrate, if the cell does not only provide the enzyme of interest, but also the machinery to generate reactive species in situ, for instance, from glucose or other bulk commodities. Finally, the plethora of small molecules present, the supramolecular organization inside the cell and competing substrate, and the reaction usage inside cells have been shown to conceivably modulate enzyme kinetics and therefore product distributions [21]. Kinetic results derived using isolated enzymes thus cannot necessarily be transferred to whole-cell catalysis.

A limitation of the use of whole-cell catalysis arises from our restricted control over intracellular concentrations and conditions, such as pH, and other ion concentrations. In addition, the intracellular chemistry is not easily followed, as a non-invasive high-resolution methodology is needed for tracking intracellular reactions. Ever-improving NMR instrumentation and methodology promise to provide increasingly detailed insight into the catalytic abilities and limitations of intact cells, including those that are widely available for bioproduction [22,23,24]. Importantly, NMR spectroscopy allows direct insights into metabolic pathways under conditions, where the cells do not necessarily thrive [25,26], as may be the case for organic synthesis with whole-cell catalysis and non-natural conditions or substrates.

In the current study, we set out to characterize the conversion of benzaldehyde and its monofluorinated isomers 2-, 3-, and 4-fluorobenzaldehyde with glucose, using commercial dry yeast (*S. cerevisiae*). The benzaldehydes/glucose substrate mixture is converted to phenylacetyl carbinols (PACs) (Scheme 1), which are precursors for ephedrine and pseudoephedrine manufacturing. Previous results have indicated that 2-fluorobenzaldehyde conversion with pyruvate and the purified *S. cerevisiae* pyruvate decarboxylase enzyme (EC 4.1.1.1) is slightly faster than conversion of benzaldehyde and its other monofluorinated variants [27]. By contrast, results from whole-cell catalysis with *S. cerevisiae* had initially suggested that 2-substituted benzaldehyde would not be well tolerated in the reaction [28], although this view was later successively revised for the small fluorine substitution [29]. Despite its promiscuous activity, some substrate selectivity can be expected for *S. cerevisiae* pyruvate decarboxylase due to the narrow crevice leading to the active site (Figure S1) [29,30], where the benzaldehyde’s access to the active site is governed especially by the bulky Ile476 [31]. Using in-cell NMR spectroscopy, we find that 2-fluorobenzaldehyde indeed is significantly preferred over benzaldehyde and the other monofluorinated variants. The high reactivity of 2-fluorobenzaldehyde at the pyruvate decarboxylase active site is consistent with the distinct steric and electronic properties elicited by the intramolecular hydrogen bonding in the substrate. Specifically, the conversion of 2-fluorobenzaldehyde to 2-fluorophenylacetyl carbinol (2F-PAC) is preferred almost tenfold over the conversion of 4-fluorobenzaldehyde to the 4-fluorophenylacetyl carbinol (4F-PAC), as measured using a competitive in-cell assay. Reaction systems can thus be devised to support significant selectivity in the conversion of isomers by *S. cerevisiae* whole-cell catalysts. Product distributions are further shown to respond to variations in cellular redox state using the addition of acetone or pyruvate. Rapid-injection spectroscopy was used to evaluate the effect on 2-fluorobenzaldehyde on cellular metabolism, showing that the 2-fluorobenzaldehyde efficiently intercepts reactive acetaldehyde at the pyruvate decarboxylase, and that 2F-PAC can be formed from extracellular substrates within a few seconds. By contrast, the flux of pyruvate to acetaldehyde, ethanol, and acetic acid are repressed by the presence of 2-fluorobenzaldehyde.

## 2. Results and Discussion

### 2.1. In-cell NMR Competition Assays of Carboligation with Benzaldehyde and Fluorinated Forms

NMR spectroscopy seems particularly well suited to the study of whole-cell catalysts and their response to competition elicited by similar substrates and to redox disturbance by additives. Specifically, we pursued the study of the acyloin coupling of glucose-derived metabolite and isomeric fluorinated benzaldehydes using in-cell NMR. *S.cerevisiae* and its pyruvate decarboxylase enzyme have been prototypic catalysts for the formation of PAC and substituted variants by means of a umpolung mechanism, as depicted in Scheme 1 [28,29,32,33,34,35]. However, the scope of isomeric substrates for intracellular competition and selectivity optimization has not been fully elucidated. We hypothesized that the complexity of intracellular metabolism with competing conceivable routes for each substrate and competing conceivable substrates for each promiscuous enzyme should afford unexpected selectivity control [36,37] that would be amenable to in-cell NMR screening and scrutiny. We set out to compare the reactivity of substrate mixtures including benzaldehyde and one mono-substituted fluorobenzaldehyde (^1^H-^13^C HSQC spectra of distilled benzaldehydes and of an equimolar mixture in Figure S2) in identical conditions, in addition to glucose and commercial dry yeast (*S. cerevisiae*). Time-course observations for the reaction were established using a D-[U-^13^C]glucose tracer to enhance the products derived from glucose ~100-fold over the natural abundance background, while providing enhanced NMR signals at characteristic chemical shifts.

**Figure 1:**
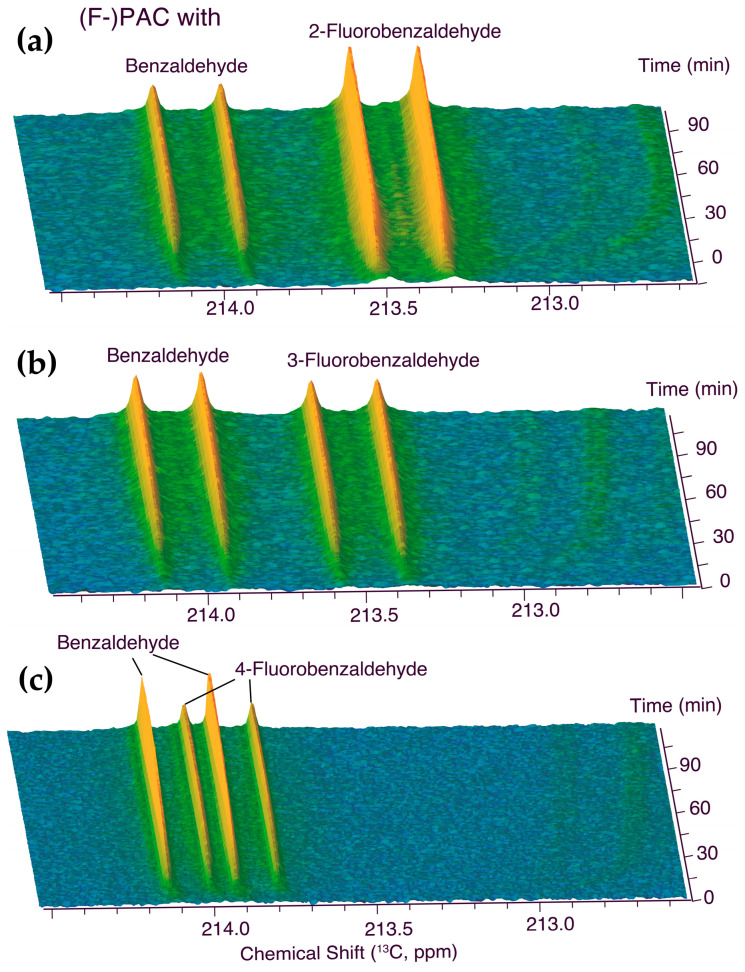
Internal competition assays between equimolar concentrations of benzaldehyde and either (**a**) 2-fluorobenzaldehyde, (**b**) 3-fluorobenzaldehyde, or (**c**) 4-fluorobenzaldehyde in *S. cerevisiae*. Reaction conditions: 0.43% (*w*/*w*) benzaldehyde and either 2-, 3-, or 4-fluorobenzaldehyde (0.5% (*w*/*w*)) with 90 mM D-[U-^13^C]glucose in 550 μL phosphate buffer (90 mM, pH 6.0, containing 1 mM MgSO_4_, 10 mM KCl, 10% D_2_O) and 100 mg commercial dry yeast, 303 K.

The keto group of the PACs is particularly well suited to probe the carboligation with α-hydroxy ketones against the cellular background, as free ketones beyond those of dihydroxyacetone phosphate and pyruvate are not prominent in cellular metabolites. Figure 1 displays the time course of the described in-cell experiment, which provided a competition assay between identical amounts of benzaldehydes and its fluorinated forms in identical conditions to determine the relative reactivities. These observations showed that 2-fluorobenzaldehyde is a significantly more reactive substrate than benzaldehyde, 3-fluorobenzaldehyde has a reactivity comparable with that of benzaldehyde, and 4-fluorobenzaldehyde is a significantly worse substrate than benzaldehyde in the formation of (fluoro-)PACs in *S. cerevisiae*. The product mixtures shown in Figure 1 were subjected to multidimensional NMR spectroscopy to validate the formation of (fluoro-)PACs. Full ^1^H and ^13^C chemical shift assignments of the fluorinated and non-fluorinated substrates, adducts, and competing products such as benzyl alcohols and benzoic acids were derived, and are compiled in the supplementary Tables S1–S4. These assignments and the experimental observations of Figure 1 indicated that the keto group of the four PACs can be distinguished using an in-cell NMR assay in a sufficiently high magnetic field (here, 800 MHz).

### 2.2. Competition in a Quinary Mixture Yields Different Results in the Cell and in Enzyme Catalysis

**Figure 2:**
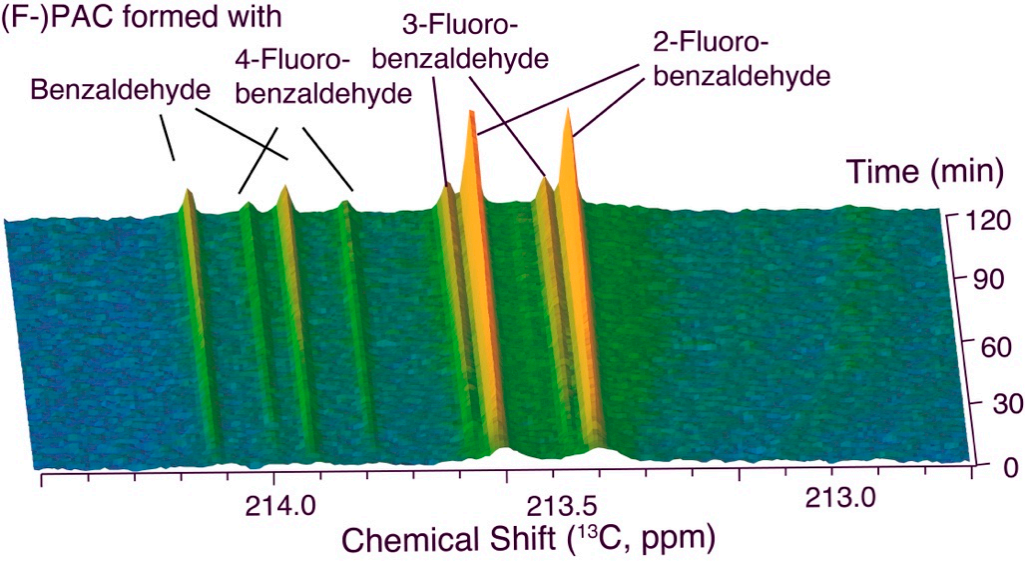
Internal competition assays between equimolar aromatic aldehydes in *S. cerevisiae*. Reaction conditions: 0.43% (*w*/*w*) benzaldehyde and 0.5% (*w*/*w*) 2-, 3-, or 4-fluorobenzaldehyde, 90 mM D-[U-^13^C]glucose in 550 μL phosphate buffer (90 mM, pH 6.0, containing 1 mM MgSO_4_, 10 mM KCl, 10% D_2_O) and 100 mg commercial dry yeast, 303 K.

We subsequently compared the reactivity of benzaldehyde and its three mono-fluorinated variants in a quinary (five-substrate) mixture with glucose. Figure 2 shows real-time observation of the competition of benzaldehyde and its three mono-fluorinated variants at equimolar concentration for activated acetaldehyde generated at the pyruvate decarboxylase cofactor thiamine diphosphate. The results from the quinary mixture, ensuring direct competition between all benzaldehydes, validated the results from the ternary reaction mixtures, as displayed in Figure 2, showing that the reactivity of the benzaldehyde monitored using in-cell NMR decreased in the order 2-fluorobenzaldehyde > 3-fluorobenzaldehyde ≈ benzaldehyde > 4-fluorobenzaldehyde. This result contrasted with initial beliefs that 2-substitued benzaldehydes would be poor substrates for in-cell PAC formation using *S. cerevisiae* due to steric conflicts at the pyruvate decarboxylase active site [29]. By contrast, the result follows some, but not all, trends reported on the isolated enzyme that indicated initial rates of conversion of 1.62, 1.36, and 1.10 for 2-fluorobenzaldehyde, 3-fluorobenzaldehyde and 4-fluorobenzaldehyde, relative to unsubstituted benzaldehyde [27]. By contrast, the real-time observations with in-cell NMR (Figure 2) indicated selectivities of 4.76, 1.19, and 0.56 for the carboligation with 2-fluorobenzaldehyde, 3-fluorobenzaldehyde and 4-fluorobenzaldehyde, relative to unsubstituted benzaldehyde.

We subsequently repeated the competition assay with commercial *S. cerevisiae* pyruvate decarboxylase and a substrate mixture containing pyruvate in addition to the same mixture of unsubstituted and monofluorinated benzaldehydes as used for whole-cell competition. This pyruvate decarboxylase catalyzed reaction indeed showed different selectivity than the whole-cell catalyzed reaction with relative selectivities 1.53, 1.17, and 0.87 (Figure S3) for the carboligation with 2-fluorobenzaldehyde, 3-fluorobenzaldehyde and 4-fluorobenzaldehyde relative to unsubstituted benzaldehyde. This observation indicated that whole-cell catalysis using competing substrates could significantly transgress the kinetic reaction control of pure enzymes. Hence, we found that whole-cell catalysis can be used to exploit intracellular biochemical logics, favoring the uptake and conversion of preferred substrates over similar substrates that enter different routes.

### 2.3. Product Composition from Whole-Cell Catalysis Exploiting Competition

**Figure 3:**
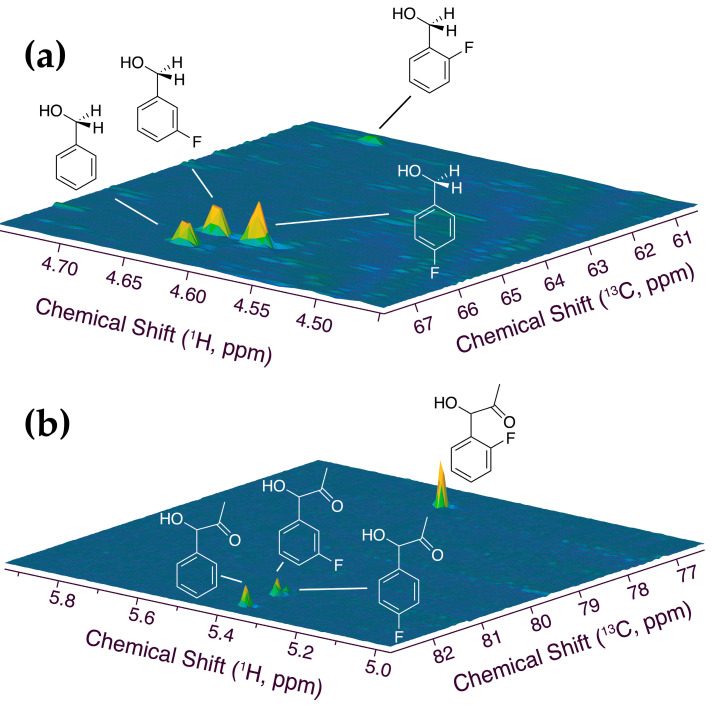
Alcohol regions of ^1^H-^13^C HSQC spectra probing the different products of benzaldehyde and its monofluorinated forms in whole-cell catalysis ((**a**) alcohols after reduction, (**b**) PACs after carboligation). Note the selective formation of 4-fluorobenzyl alcohol and 2F-PAC. Reaction conditions: 0.43% (*w*/*w*) benzaldehyde and 0.5% (*w*/*w*) 2-, 3-, or 4-fluorobenzaldehyde, 90 mM glucose in 550 μL phosphate buffer (90 mM, pH 6.0, containing 1 mM 1 mM MgSO_4_, 10 mM KCl, 10% D_2_O) and 100 mg commercial dry yeast, 303 K, 1 h reaction time.

**Figure 4:**
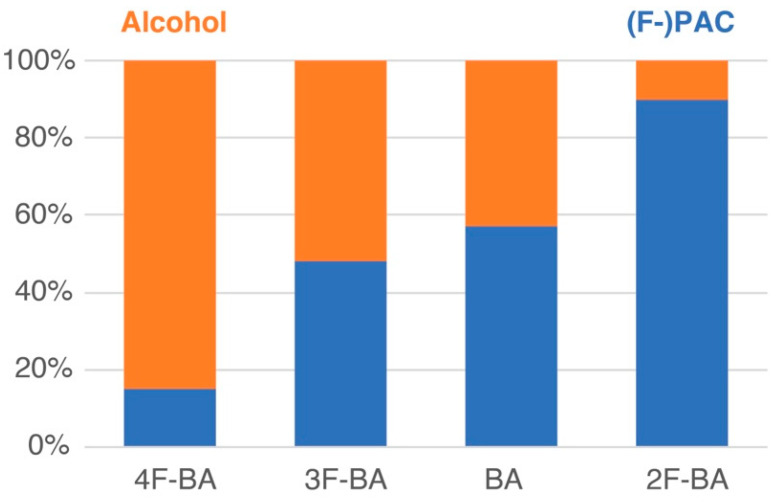
Product distribution in the post-reaction mixture of Figure 3, showing selective formation of 4-fluorobenzyl alcohol and 2F-PAC.

The different fate of the similar substrates was assessed through suitably adapted high-resolution ^1^H-^13^C HSQC assays [38,39,40,41,42] to determine the product composition resulting from the conversion of unsubstituted and monofluorinated benzaldehydes (Figure 3). Full conversion of the benzaldehydes was achieved at sufficiently low concentrations of the benzaldehydes, thus raising the question of how the distribution between PAC and other products (especially benzyl alcohols) differs, considering the high selectivity of 2-fluorobenzaldehyde conversion to its PAC. Figure 3 shows the ^1^H-^13^C HSQC spectral region of alcohol groups in PACs and the benzyl alcohol forms resulting from equimolar mixtures of unsubstituted and monofluorinated benzaldehydes. An anticorrelation was directly evident in ^1^H-^13^C HSQC, showing that the trend for PAC-formation is reversed in benzyl alcohol formation to yield selectivities that increase 2-fluorobenzaldehyde < 3-fluorobenzaldehyde ≈ benzaldehyde < 4-fluorobenzaldehyde for substrate reduction. Product distributions were derived from ^1^H-^13^C HSQC signal volumes, yielding the product distributions of Figure 4. These distributions indicated that high selectivity can be achieved in the conversion of isomeric 2-fluorobenzaldehyde and 4-fluorobenzaldehyde in substrate mixtures converted via whole-cell catalysis, the former favoring adduct formation to the 2F-PAC with a selectivity of 90%, and the latter favoring reduction to 4-fluorobenzyl alcohol. The high reactivity of 2-fluorobenzaldehyde at the pyruvate decarboxylase active site indicates the role of the intramolecular hydrogen bond to the aldehyde group (leading to a distinctly deshielded ^1^H chemical shift in 2-fluorobenzaldehyde; Tables S1–S4) in restraining the conformational flexibility of the substrate to meet steric requirements at the enzyme active site.

**Figure 5:**
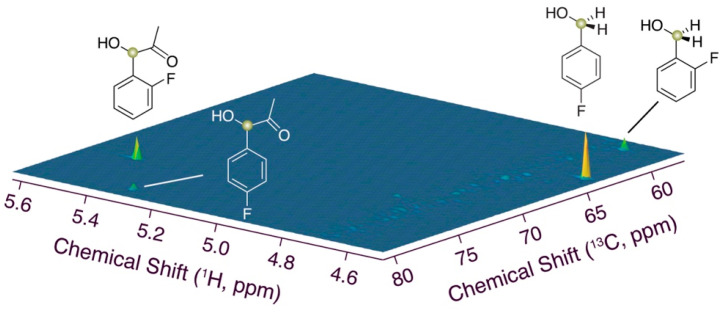
Alcohol region of ^1^H-^13^C HSQC spectra probing the different products, 2-fluorobenzaldehyde and 4-fluorobenzaldehyde. Reaction conditions: 0.5% (*w*/*w*) 2-fluorobenzaldehyde and 0.5% (*w*/*w*) 4-fluorobenzaldehyde, 90 mM glucose in 550 μL phosphate buffer (90 mM, pH 6.0, containing 1 mM MgSO_4_, 10 mM KCl, 10% D_2_O), 100 mg commercial dry yeast, 303 K, 1 h reaction time.

To validate the possibility of constructing substrate mixtures based on competitive assays to obtain improved selectivities and yields with whole-cell catalysis, we simplified the substrate mixture to a ternary mixture of 2-fluorobenzaldehyde and 4-fluorobenzaldehyde reacted with glucose and *S. cerevisiae*. The resultant ^1^H-^13^C HSQC spectrum using the alcohol groups as probes of product composition is shown in Figure 5. This spectrum validates the remarkable selectivity (~90%) in the conversion of 2-fluorobenzaldehyde to 2F-PAC as a fluorinated precursor of (pseudo-)ephedrine and the concurrent highly selective reduction of 4-fluorobenzaldehyde in suitably designed reaction mixtures. By contrast, assays using 2-fluorobenzaldehyde (0.5% *w*/*w*) alone rather than the fluorobenzaldehyde mixture yielded mixtures of 57%(±6%) 2F-PAC and 43% 2-fluorobenzyl alcohol. The better selectivity towards 2F-PAC in the presence of sacrificial 4-fluorobenzaldehyde appears to result from a stronger influx of 2-fluorobenzaldehyde into PACs, and of 4-fluorobenzaldehyde into alcohol in the substrate mixture.

### 2.4. Competition with Natural Acetaldehyde Clearance and NADH Oxidation

**Figure 6:**
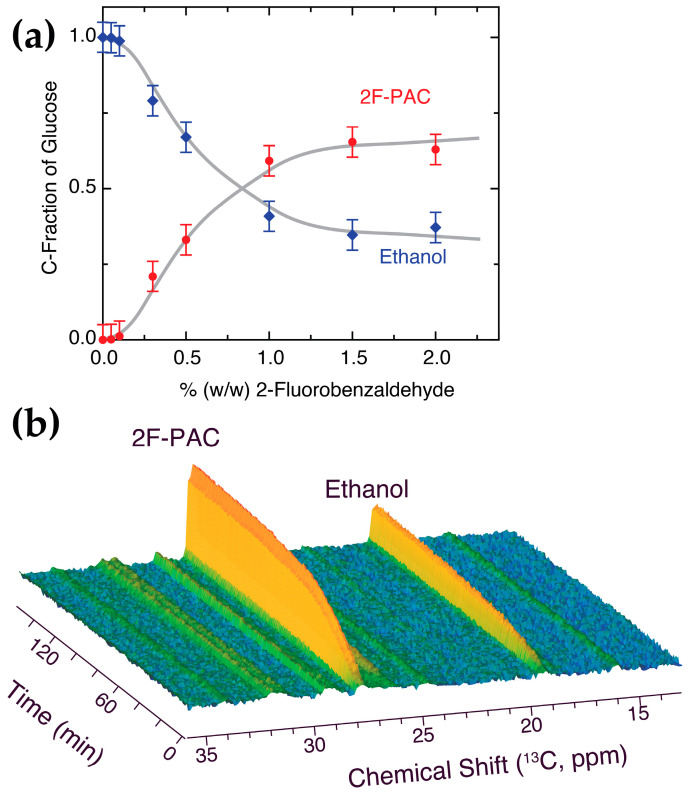
(**a**) Internal conversion of glucose-derived acetaldehyde to ethanol or 2F-PAC, independent of the weight % of 2-fluorobenzaldehyde. The expected dependence on 2-fluorobenzaldehyde concentration shows a massive redirecting of glucose carbon from alcoholic fermentation to 2F-PAC formation. Reaction conditions: variable weight-% 2-fluorobenzaldehyde, 90 mM glucose in 550 μL phosphate buffer (90 mM, pH 6.0, containing 1 mM MgSO_4_, 10 mM KCl, 10% D_2_O), 100 mg commercial dry yeast, 303 K, 1 h reaction time. (**b**) Real-time spectroscopic assay of 2F-PAC formation in comparison to ethanol formation. Reaction conditions: 2.8 % (*w*/*w*) 2-fluorobenzaldehyde, 90 mM D-[U-^13^C]glucose, 550 μL phosphate buffer (90 mM, pH 6.0, containing 1 mM MgSO_4_, 10 mM KCl, 10% D_2_O), 100 mg commercial dry yeast, 303 K.

Subsequently, we expanded the study of the competition between benzaldehyde derivatives to a study of the competition between non-evolved and evolved pathways in whole-cell catalysis. The high selectivity that can be achieved in the competition between substituted benzaldehyde raises questions about interference of non-natural benzyl alcohol formation and carboligation in the natural (evolved) function of the cell in pyruvate decarboxylation to acetaldehyde and NADH regeneration through the reduction of acetaldehyde to ethanol. Fluorobenzaldehyde isomers can both sequester acetaldehyde and replace it as the oxidant of NADH. Hence, we pursued a comparison of glucose carbon flux through glycolysis to ethanol and to the 2F-PAC adduct in the presence of varying amounts of 2-fluorobenzaldehyde (Figure 6) by comparing 2F-PAC and ethanol formation.

The results of Figure 6a show that most of the glucose carbon is converted to 2-fluorophenylacetyl carbinol at concentrations of 2-fluorobenzaldehyde above 1% (*w*/*w*) in the reaction mixture. The fraction of glucose carbon entering the non-evolved pathway can thus significantly exceed the fraction of glucose entering the evolved pathway to ethanol. This notion was subsequently validated by real-time in-cell observations of the competition between natural and non-natural pathways, as shown in Figure 6b at 3% (*w*/*w*) 2-fluorobenzaldehyde. This real-time observation validates that approximately 75% non-natural usage of glucose carbon is viable in the notoriously ethanol-forming *S. cerevisiae* through interception of activated acetaldehyde at the pyruvate decarboxylase with xenochemicals. Hence, various NMR assays elucidating the competition between xenochemicals and between natural and non-natural pathways in living yeast highlight the versatility of its metabolism [43], and the drastic consequences for its metabolism in the presence of non-natural chemicals [25,44,45]. Consistent with the in-cell competition assays described above, higher concentrations of benzaldehyde (2%) were necessary to intercept most acetaldehyde from its conversion to ethanol (Figure S4).

### 2.5. Mechanistic Effect of Xenochemicals on Natural Biochemistry Using D-DNP NMR

Additional detail on 2F-PAC formation could be obtained using D-DNP NMR spectroscopy to assess changes in central carbon metabolism, owing to the presence of 2-fluorobenzaldehyde. D-DNP NMR can use a glucose tracer with an approximately 20,000-fold enhanced nuclear spin polarization [46] to visualize the real-time flux of glucose molecules through glycolysis on the seconds timescale [23,47,48,49], and can render kinetic bottlenecks amongst the glycolytic intermediates detectable.

**Figure 7:**
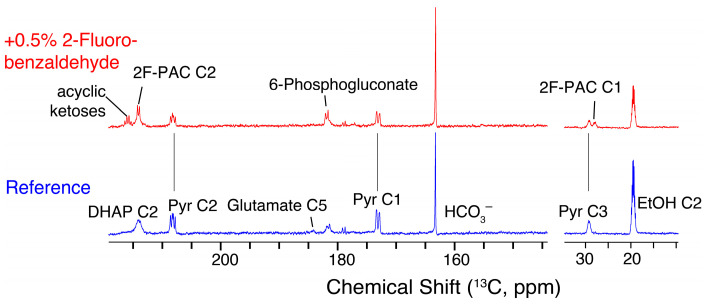
Sum spectra of a D-DNP time series showing the influx of hyperpolarized D-[U-^13^C,U-^2^H]glucose into metabolites within 30 s in the presence of 0.5% (*w*/*w*) 2-fluorobenzaldehyde (red) and in its absence. 2F-PAC forms rapidly, and its effects on other metabolites, including metabolites of the pentose phosphate pathway, glyceronephosphate (DHAP), pyruvate (Pyr), and ethanol (EtOH), are discernible.

Figure 7 shows ^13^C NMR spectra obtained for *S. cerevisiae* glycolysis in the presence and in the absence of 2-fluorobenzaldehyde to expand beyond the observation of reduced ethanol formation. The spectra of Figure 7 show, which metabolites accumulate from glucose within 30 s upon pulse feeding with hyperpolarized glucose in the absence (reference, blue) and in the presence (red) of 0.5% (*w*/*w*) 2-fluorobenzaldehyde (time-resolved data in the presence of 2-fluorobenzaldehyde are shown in Figure S5). The experiments validate that ethanol formation drops in the presence of 2-fluorobenzaldehyde, and that 2-fluorobenzaldehyde efficiently intercepts activated acetaldehyde, as the 2F-PAC signal emerges within seconds from the hyperpolarized glucose signal. Pyruvate accumulates less in the presence of 2-fluorobenzaldehyde than in its absence, possibly due to faster recovery of free pyruvate decarboxylase in the presence of an alternative pathway for the conversion of activated acetaldehyde. Indication of glucose tracer influx into glutamate is only found in the absence of 2-fluorobenzaldehyde (signal near 184 ppm), thus underlining the increased competition for activated acetaldehyde in the presence of 2-fluorobenzaldehyde. Differences between both experiments also show that the presence of 2-fluorobenzaldehyde alters fluxes upstream of the pyruvate decarboxylase. Most notoriously, the influx of glucose into acyclic ketose signals (DHA, ribulose-5-phosphate, and xylulose-5-phosphate) and 6-phosphogluconate indicates enhanced influx into the oxidative pentose phosphate pathway in the presence 2-fluorobenzaldehyde. By contrast, DHAP accumulates less in the presence of 2-fluorobenzaldehyde, possibly due to its faster conversion, because of the higher NAD^+^ availability afforded by the exogeneous aldehyde. Hence, D-DNP observations overall indicate that benzaldehyde affects metabolism in the whole-cell catalyst, both by competition at the pyruvate decarboxylase step and by affecting the intracellular redox state.

### 2.6. Effect of Oxidized Additive and Substrate

**Figure 8:**
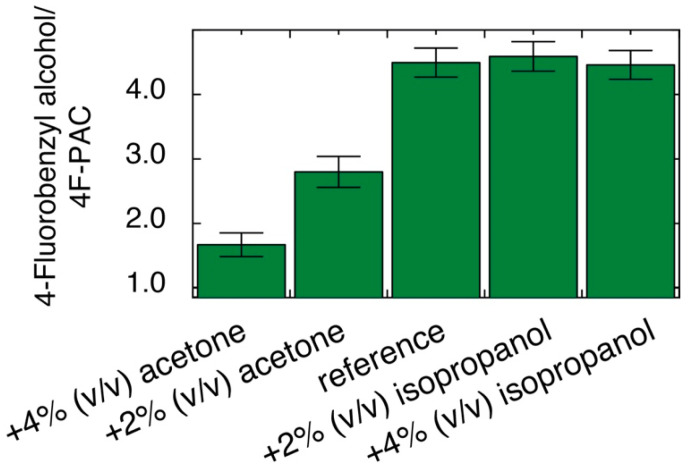
Effect of acetone and isopropanol on product distribution from 4-fluorobenzaldehyde in *S. cerevisiae*. Reaction conditions: 0.5% (*w*/*w*) 4-fluorobenzaldehyde, 90 mM glucose, 550 μL phosphate buffer (90 mM, pH 6.0, containing 1 mM MgSO_4_, 10 mM KCl, 10% D_2_O), 100 mg commercial dry yeast, 303 K, 1 h.

Considering the interplay between 2-fluorobenzaldehyde and cellular physiology, including the cellular redox state, we finally evaluated if cellular physiology can be used to affect the selectivity in the conversion of fluorobenzaldehydes to fluorinated PACs and fluorobenzyl alcohols. Initially, we evaluated if modulation of the cellular redox state by the presence of acetone or isopropanol had a systematic effect on the conversion of fluorobenzaldehyde with glucose. Acetone has previously been used to oxidize NADH, while isopropanol has been used to reduce NAD^+^ in yeast to affect redox reactions on xenochemicals [12]. Figure 8 shows that the presence of acetone affected the cellular tendency to reduce aldehyde and led to decreased selectivity in the formation of 4-fluorobenzyl alcohol from 4-fluorobenzaldehyde. Specifically, the ratio between 4-fluorobenzyl alcohol and 4F-PAC was decreased threefold by the presence of 4% (*v*/*v*) acetone. Additionally, the selectivity of 2-fluorobenzaldehyde towards 2F-PAC rather than 2-fluorobenzyl alcohol could be further increased by the presence of acetone (Figure S6). By contrast, the presence of up to 4% (*v*/*v*) isopropanol in our model reaction had minor effects. Accordingly, acetone emerged as a useful additive to favor carbinol formation, relative to alcohol formation, thus leading to a more equal distribution between 2F-PAC and 4F-PAC formed in competition from an equimolar substrate mixture.

**Figure 9:**
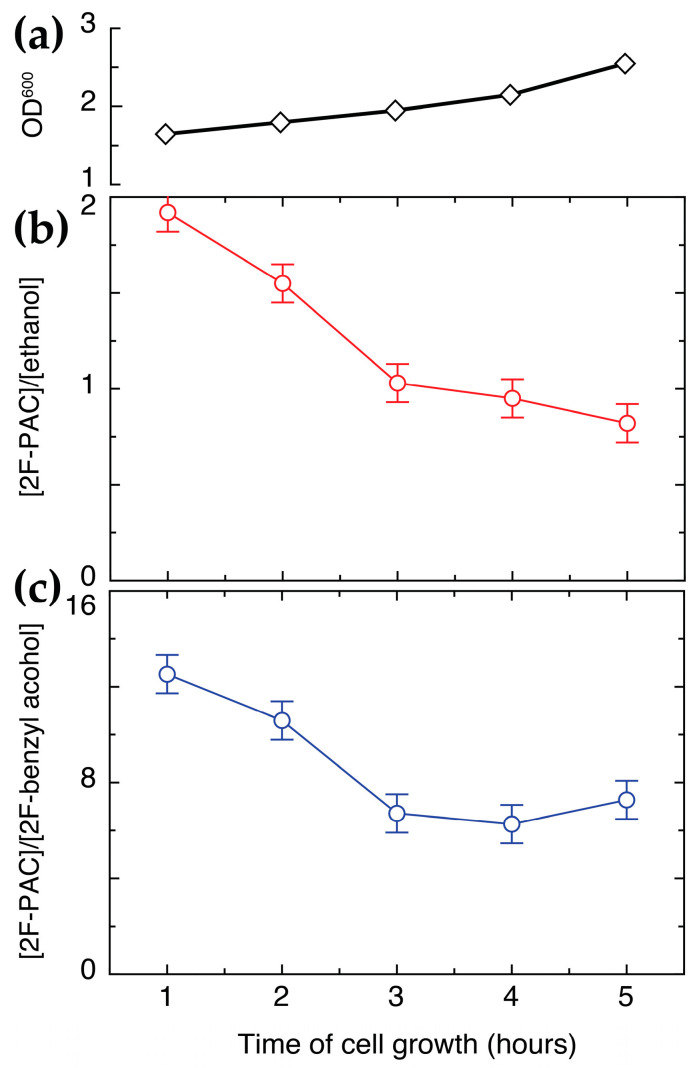
The effect of cell growth (**a**) on the conversion of pyruvate and product distribution in the reaction between 2-fluorobenzaldehyde and pyruvate. (**b**) The ratio between 2F-PAC and ethanol as a function of growth time prior to the reaction. (**c**) The ratio between 2F-PAC and 2-fluorobenzyl alcohol as a function of growth time prior to the reaction. The results indicate that pyruvate favors 2F-PAC formation, and that the effect fades with fading pyruvate uptake in growing cell cultures. Reaction conditions: pre-incubation of 100 mg commercial dry yeast in YPD for variable time, subsequent reaction of 0.5% (*w*/*w*) 2-fluorobenzaldehyde, 180 mM pyruvate, identical number of cells in 550 μL phosphate buffer (90 mM, pH 6.0, containing 1 mM MgSO_4_, 10 mM KCl, 10% D_2_O), 303 K, 1 h.

Encouraged by the significant effect of oxidant on the selectivity for 2-PAC and 4-PAC formation, we finally evaluated if an oxidized metabolite could be used as a directing agent towards PAC formation, and to this end, we chose pyruvate. Pyruvate can replace glucose as the precursor of activated acetaldehyde in the formation of PAC. In addition, we wanted to shed light on the hypothesis that pyruvate loses its suitability as a carbon source in whole-cell catalysis during yeast growth, possibly due to a preferred usage of glucose in thriving cells [12]. We therefore incubated *S. cerevisiae* in YPD medium for variable time (1–5 h) prior to reacting pyruvate (180 mM) and 2-fluorobenzaldehyde with a defined number of these cells in different growth phases. The findings were compared to a reaction conducted with glucose (90 mM) and 2-fluorobenzaldehyde. The results are shown in Figure 9 and Figure S7. The data were consistent, showing an effect of pyruvate on the cellular redox state that favored the formation of 2F-PAC over alcohol and 2-fluorobenzyl alcohol initially, but less so for growing cells. The preference for 2F-PAC formation over alcohol and over 2-fluorobenzyl alcohol formation from pyruvate and 2-fluorobenzaldehyde decreased after the growth of cells in YPD medium, approaching the distribution observed for the reaction between glucose and 2-fluorobenzaldehyde. These observations indicated that dry yeast was a better catalyst of 2-fluorobenzaldehyde’s conversion with pyruvate, while the effect of cell growth on the reaction with glucose was negligible and possibly slightly positive (Figure S7). Overall, the results showed that carboligation on 2-fluorobenzaldehyde proceeds better in the presence of an oxidized carbon source (pyruvate) or additive (4-fluorobenzaldehyde, acetone).

## 3. Materials and Methods

### 3.1. Chemicals

Benzaldehyde (>99%) and 2-fluorobenzaldehyde (>98%) were purchased from Thermo Scientific (Waltham, MA, USA), while 3-fluorobenzaldehyde (>97%) and 4-fluorobenzaldehyde (>98%) were purchased from TCI Europe (Zwijndrecht, Belgium). All benzaldehyde derivates proved to contain benzyl alcohol and benzoic acid forms after opening, which was sufficient to achieve full ^1^H and ^13^C chemical shift assignments for unsubstituted and monofluorinated benzaldehydes, benzoic acids, and benzyl alcohols (Tables S1–S4). Uniformly ^13^C-enriched D-glucose (D-[U-^13^C]glucose) and uniformly ^13^C and deuterium-enriched D-glucose (D-[U-^13^C,U-^2^H]glucose) were obtained from Sigma Aldrich (Andover, MA, USA), as were NaH_2_PO_4_·H_2_O, KCl, MgSO_4_, sodium pyruvate, *S. cerevisiae* pyruvate decarboxylase, D-glucose at natural isotopic abundance, isopropanol, acetone, and D_2_O.

### 3.2. Distillation

A round-bottomed flask containing benzaldehyde or its fluorinated derivatives was placed on a thoroughly cleaned rotary evaporator with an applied vacuum of 25–30 mbar, and the temperature of the water bath set to 90 °C. The benzaldehydes were distilled into a freshly cleaned reservoir and then transferred to a flame-dried round-bottomed flask for degassing (N_2_-bubbling). The freshly distilled and degassed benzaldehydes were used immediately and were stored at 4 °C under N_2_ atmosphere. Distillation was conducted to warrant well-defined amounts of the benzaldehydes and to avoid cytotoxicity of benzoic acids. Stock solutions in DMSO-d6 (10% *w*/*w*) were used to ensure the reliable delivery of the hydrophobic substrates to catalytic assays. Growth experiments in the presence of up to 0.5% (*w*/*w*) (fluoro-)benzaldehydes validated that these concentrations are not prohibitive to cell growth; these results are consistent with those of initial studies on yeast viability in the presence of benzaldehydes [28,35].

### 3.3. Assignment of Phenylacetyl Carbinol, including Monofluorinated Forms

Distilled benzaldehydes (0.5% (*w*/*w*) fluorobenzaldehydes and 0.43% (*w*/*w*) benzaldehyde) were incubated with 90 mM glucose in 550 μL phosphate buffer (90 mM, pH 6.0, containing 1 mM MgSO_4_, 10 mM KCl, 10% D_2_O) and 100 mg commercial dry yeast (*S. cerevisiae*, Seitenbacher, Germany) for 60 min under vigorous shaking (600 rpm) at 303 K in a DITABIS Heating-ThermoMixer. Subsequently, the reaction mixtures were centrifuged, and ^1^H-^13^C HSQC and ^1^H-^13^C HMBC spectra were acquired from the four reaction mixtures using a Bruker (Fällanden, Switzerland) 800 MHz NMR instrument equipped with a TCI cryoprobe and a SampleJet sample changer. The ^1^H-^13^C HSQC spectra were acquired by collecting 2048 × 512 complex data points (in the direct and indirect dimension, respectively) sampling 213 ms and 15 ms, respectively. The ^1^H-^13^C HMBC spectra were acquired by sampling 2048 × 256 complex data for 213 ms and 15 ms, respectively. The heteronuclear assignment spectra were analyzed in Bruker Topspin 4.1.4 to yield the assignment spectra of unsubstituted and monofluorinated PAC, as provided in Tables S1–S4.

### 3.4. Competition Assay

Internal competition assays were initially conducted by reacting mixtures containing 0.43% (*w*/*w*) benzaldehyde and either 2-, 3-, or 4-fluorobenzaldehyde (0.5% (*w*/*w*)) with 90 mM D-[U-^13^C]glucose in 550 μL phosphate buffer (90 mM, pH 6.0, containing 1 mM MgSO_4_, 10 mM KCl, 10% D_2_O) and 100 mg commercial dry yeast. The tendencies of the monofluorinated benzaldehydes to form monofluoro-PACs were compared to the tendency of benzaldehyde to form PAC with intact *S. cerevisiae* cells by assessing product formation using a series of ^13^C NMR spectra acquired with a Bruker 800 MHz NMR instrument equipped with a TCI cryoprobe and 18.7 T Oxford magnet. To this end, a time series of ^13^C NMR spectra (16384 complex data points) was acquired using 30° flip angle excitation pulses and inverse gated decoupling of ^1^H by accumulating 16 transients with an interscan relaxation delay of 1.5 s; this was achieved by sampling the FID for 0.68 s.

Subsequently, the internal competition reaction was repeated using 0.43% (*w*/*w*) benzaldehyde and 0.5% (*w*/*w*) each of 2-, 3-, or 4-fluorobenzaldehyde with 90 mM D-[U-^13^C]glucose in 550 μL phosphate buffer (90 mM, pH 6.0, containing 1 mM MgSO_4_, 10 mM KCl, 10% D_2_O) and 100 mg commercial dry yeast. The reaction was again followed by a series of ^13^C NMR spectra, validating the relative reactivities of benzaldehyde and monofluorobenzaldehyde obtained from their binary mixtures.

The competition of 0.43% (*w*/*w*) benzaldehyde and 0.5% (*w*/*w*) each of 2-, 3-, or 4-fluorobenzaldehyde (equimolar mixture) with activated acetaldehyde was subsequently repeated for the enzyme-catalyzed reaction using the same mixture as for whole-cell catalysis in 550 μL phosphate buffer (90 mM, pH 6.0, containing 1 mM MgSO_4_, 10 mM KCl, 10% D_2_O) and 150 mM pyruvate, as well as 1 unit commercial *S. cerevisiae* pyruvate decarboxylase enzyme.

### 3.5. ^1^H-^13^C Assay of Distribution between Benzyl Alcohol and Phenylacetyl Carbinol Formation

A mixture of 0.43% (*w*/*w*) benzaldehyde and 0.5% (*w*/*w*) each of 2-, 3-, or 4-fluorobenzaldehyde (equimolar mixture) or a mixture of 0.5% (*w*/*w*) each of 2- and 4-fluorobenzaldehyde was incubated with 90 mM glucose in 550 μL phosphate buffer (90 mM, pH 6.0, containing 1 mM MgSO_4_, 10 mM KCl, 10% D_2_O) and 100 mg commercial dry yeast for 60 min under vigorous shaking (600 rpm) at 303 K. Subsequently, reaction mixtures were centrifuged, and supernatants were subjected to ^1^H-^13^C HSQC analysis of the respective (fluoro-)benzyl alcohol and (fluoro-)PAC signals, which were identified from the assignments of Tables S1–S4 and quantified using responses relative to a fully relaxed ^1^H-^13^C HSQC acquired with an interscan relaxation delay of 20 s [50,51]. The reaction containing 0.5% (*w*/*w*) each of 2- and 4-fluorobenzaldehyde was further repeated and analyzed in the presence of either isopropanol or acetone at 2% (*v*/*v*) or 4% (*v*/*v*).

### 3.6. Effect of Growth Phase and of Pyruvate As a Carbon Source

A pre-incubation was conducted by incubating 100 mg dry yeast in 50 mL YPD medium for 1–5 h prior to measurement of the OD^600^ and harvest of identical cell numbers by centrifugation for 3 min at room temperature (OD^600^ × Volume = 82.5 mL). This was followed by the discarding of the supernatant, resuspension in 2.5 mL phosphate buffer (90 mM, pH 6.0, containing 1 mM MgSO_4_, 10 mM KCl) for washing, and centrifugation for 3 min. The cell pellet was resuspended in a mixture of 0.5% (*w*/*w*) 2-fluorobenzaldehyde (using a 10% (*w*/*w*) solution in DMSO-d_6_) and 180 mM pyruvate in 550 μL phosphate buffer (90 mM, pH 6.0, containing 1 mM MgSO_4_, 10 mM KCl, 10% D_2_O) under vigorous shaking (600 rpm) at 303 K for 1 h. Reaction mixtures were centrifuged, and supernatants were analyzed using ^1^H-^13^C HSQC NMR.

### 3.7. Hyperpolarization

Hyperpolarization of D-[U-^13^C,U-^2^H]glucose for subsequent D-DNP NMR was performed according to a previously established protocol [47,52]; solid-state dynamic nuclear polarization was conducted by mixing D-[U-^13^C,U-^2^H]glucose with trityl radical OX063 (27 mM; Oxford Instruments, Abingdon, UK) and gadoteridol (1.5 mM; Bracco Imaging, Italy) in ultra-pure water. The final substrate samples contained 0.05 mmol D-[U-^13^C,U-^2^H]glucose in 19 mg of the sample preparation. The preparation was placed in a sample cup for hyperpolarization and transferred to a Hypersense polarizer operating at 1.2 K and using microwave irradiation at 94.049 GHz with 100 mW in a magnetic field of 3.35 T. After 1 h, solid-state polarizations above 30% were obtained.

### 3.8. D-DNP NMR

Upon hyperpolarization with DNP in a Hypersense polarizer, the preparation was dissolved with heated phosphate buffer containing 10 mM KCl and 1 mM MgSO_4_ (5 mL of 90 mM buffer, pH 6.0). In this manner, hyperpolarized samples with a final substrate concentration of 11 mM D-[U-^13^C,U-^2^H]glucose were obtained. Of these hyperpolarized samples, 0.33 mL were injected into 0.2 mL of yeast cell suspension in YNB in the absence or in the presence of distilled 2-fluorobenzaldehyde (final concentration 0.5% (*w*/*w*)), equilibrated to 303 K inside a 500 MHz Bruker spectrometer equipped with a 5 mm DCH CryoProbe and an 11.7 T UltraShield magnet. This procedure yielded a final concentration of 6.8 mM hyperpolarized glucose in the NMR tube. Reactions were followed using a series of ^13^C NMR spectra acquired with ∼12° excitation pulses as pseudo-2D spectra, using a receiver gain of 10 to acquire the ^13^C NMR spectra of 11,264 complex data points (sampling the ^13^C FID for 344.7 ms) every 0.5 s.

## 4. Conclusions

The current study sought insight into the ability to convert isomeric non-natural substrates into diverse products in whole-cell catalysis, which is akin to the effects that cellular biochemistry often has on chemically similar natural substrates. We find that competing, chemically similar non-natural substrates can affect the reaction selectivity inside the cell, leading to different results than those expected from purified enzyme catalysts. Screening using in-cell NMR spectroscopy allowed us to explore the interaction between substrates and to design selective transformations. Notably, non-biological atoms can be installed into the products in this manner, which may lead to altered biological activity and stability in the products. We found that fluorine in isomeric xenochemicals may act as a successful directing group, as 2-fluorobenzaldehyde outcompetes 4-fluorobenzaldehyde at the pyruvate decarboxylase step for carboligation with activated acetaldehyde. By contrast, 4-fluorobenzaldehyde outcompetes 2-fluorobenzaldehyde for reduction to the benzyl alcohol. In combination, selectivities near 90% could be achieved for the conversion of 2-fluorobenzaldehyde and 4-fluorobenzaldehyde with glucose and yeast to 2F-PAC and 4-fluorobenzyl alcohol. These findings question the view that higher selectivity usually justifies the higher catalyst cost of purified enzymes, as the versatility of whole-cell catalysis seems underexplored. Future work tailoring the active sites of complementary enzyme pairs through engineering or evolutionary approaches for whole-cell conversion of selected substrate pairs thus seems both desirable and promising.

Beyond encouraging the use of carefully designed substrate mixtures with competing isomers, oxidized additives, and oxidized carbon sources for yeast in whole-cell catalysis, the findings indicate the evolutionary strategies of microorganisms to deal with small chemicals in nature; *S. cerevisiae* employs concurrent pathways to react free aldehydes with more stable functional groups, either through C-C bond formation or through reduction. Reactions with the non-natural aromatic aldehydes reflect natural strategies to clear reactive metabolites through C-C bond formation or reduction. Oxidation was no significant factor in the conversion of benzaldehydes, possibly as NAD^+^ is maintained for the oxidative initial steps in glycolysis and the pentose phosphate pathway. D-DNP NMR was used to shed light on the interplay between benzaldehyde xenochemicals and natural yeast metabolism, showing effects on pathway kinetics and usage throughout central carbon metabolism.

Many cross interactions occur between small molecules and enzymes inside the cell, and selectivity can be affected by localization, supramolecular enzyme organization, and competition [53,54]. Overall, this study indicates that the (sometimes unexpected) interplay between reactants in bio-based carboligation using whole-cell catalysis can be explored more routinely and utilized more rationally based on noninvasive observations.

## Data Availability

Not applicable.

## Supplementary Materials

The following supporting information can be downloaded at: https://www.mdpi.com/article/10.3390/molecules28135157/s1, Figure S1: Structural representation of *S. cerevisiae* pyruvate decarboxylase; Figure S2: Aromatic region of ^1^H-^13^C HSQC spectra of distilled benzaldehydes and of the equimolar mixture; Figure S3: Formation of PACs from an equimolar mixture of unsubstituted and monofluorinated benzaldehydes and *S. cerevisiae* pyruvate decarboxylase; Figure S4. Internal competition of glucose-derived acetaldehyde for ethanol or PAC formation, independent of the weight % of benzaldehyde; Figure S5. D-DNP NMR time series; Figure S6. The effect of acetone and isopropanol on product distribution from 2-fluorobenzaldehyde in *S. cerevisiae*. Figure S7. The effect of cell growth and uptake of oxidized reactant pyruvate on product distribution in the reaction between 2-fluorobenzaldehyde and glucose; Table S1. Full ^1^H and ^13^C chemical shift assignments of benzaldehyde and its products; Table S2. Full ^1^H and ^13^C chemical shift assignments of 2-fluorobenzaldehyde and its products; Table S3. Full ^1^H and ^13^C chemical shift assignments of 3-fluorobenzaldehyde and its products; Table S4. Full ^1^H and ^13^C chemical shift assignments of 4-fluorobenzaldehyde and its products.
