# Supplementary material for: Conversion of Similar Xenochemicals to Dissimilar Products: Exploiting Competing Reactions in Whole-Cell Catalysis
Source: Molecules. 2023 Jul 1;28(13):5157. doi: 10.3390/molecules28135157 (PMC10343759; doi:10.3390/molecules28135157)
Supplement: Supplementary file 1 [file molecules-28-05157-s001.zip › molecules-2451219-supplementary.pdf]

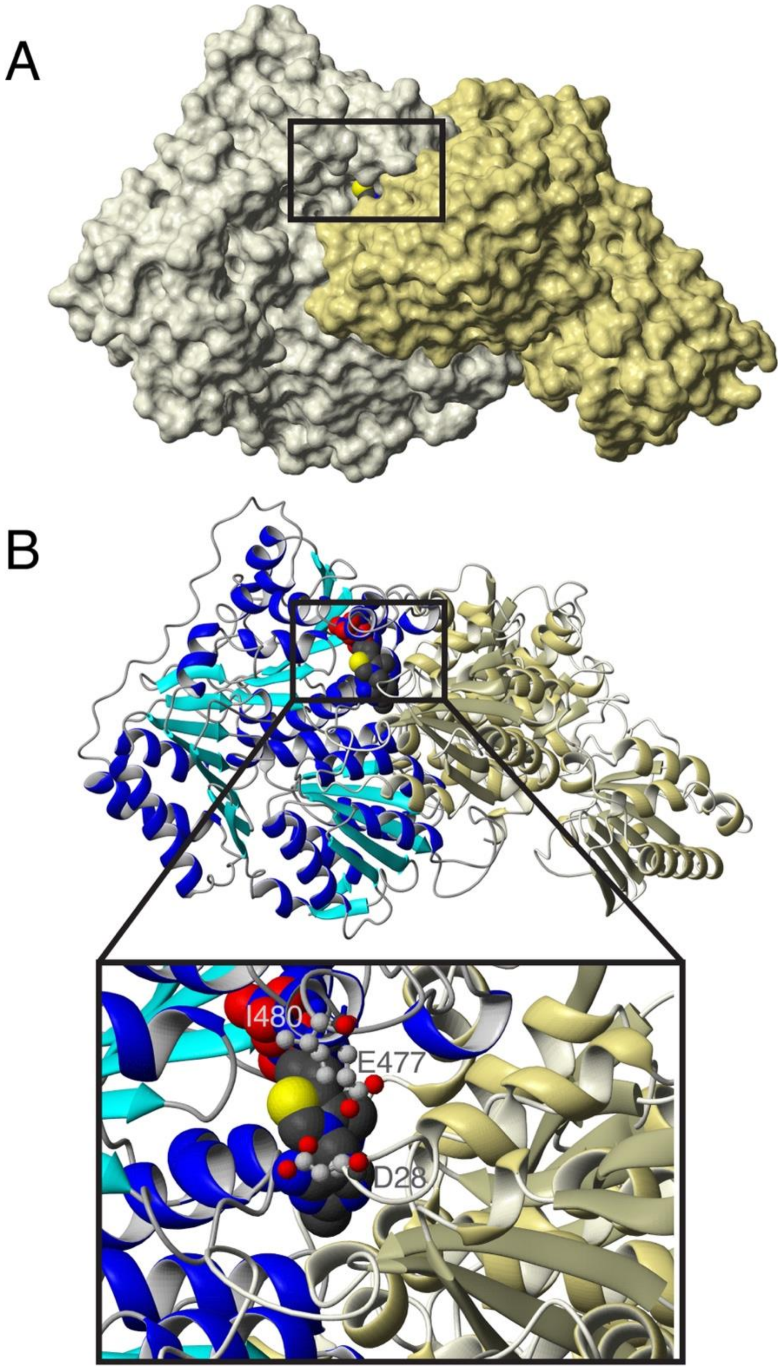

Figure S1. (A) Surface representation of monomer interface in *S. cerevisiae* pyruvate decarboxylase (EC 4.1.1.1) (coordinates from a 2.3 Å crystal structure; PDB code 1PVD). Two monomers are in the asymmetric unit, while the protein occurs as a dimer of dimers, with the active site located at the end of a narrow crevice between two monomers (highlighted by a black frame). (B) Ribbon representation in the same orientation, with a zoom onto the active site and some flanking amino acids (D28 from a neighboring monomer). The thiamine diphosphate cofactor is shown in space filling representation.

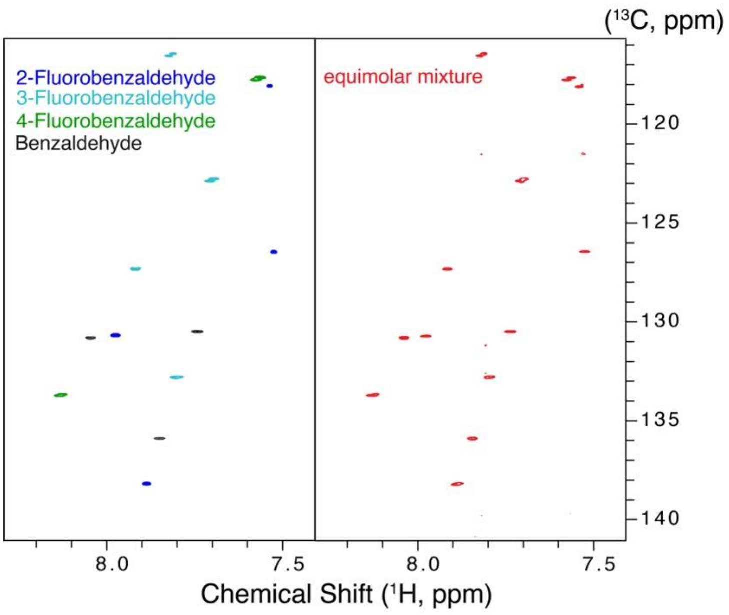

Figure S2. Aromatic region of ^{1}H-^{13}C HSQC spectra of distilled benzaldehyde and of 2-, 3-, or 4-fluorobenzaldehyde shown in overlay (left) as well as an equimolar quaternary mixture of all four aromatic aldehydes used for competition assays (right).

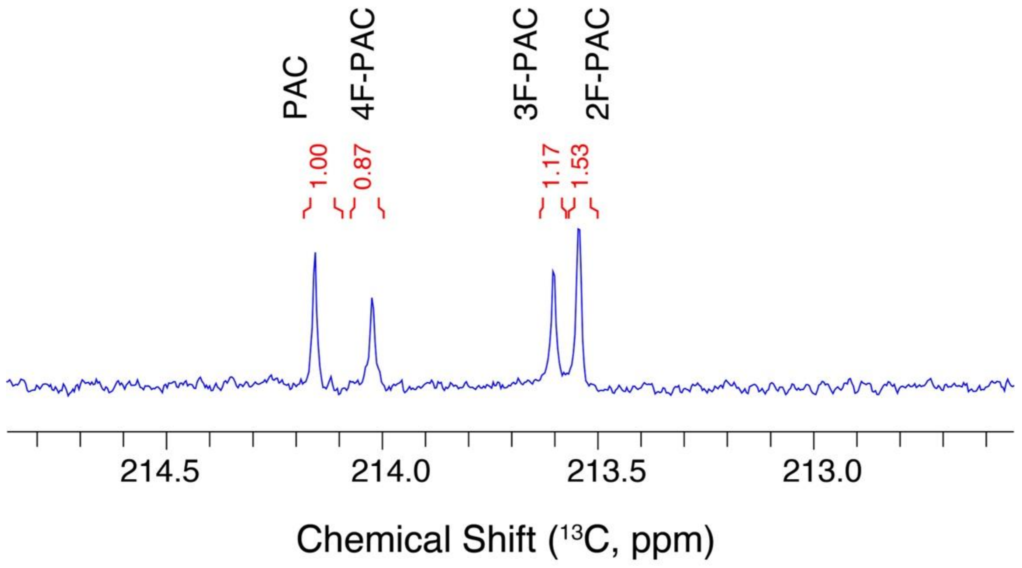

Figure S3. Formation of PACs from an equimolar mixture of unsubstituted and monofluorinated benzaldehydes and 1 unit of *S. cerevisiae* pyruvate decarboxylase, showing altered selectivity relative to the use of whole cell catalysis in intracellular competition. Reaction conditions: 0.43% (w/w) of benzaldehyde and 0.5 % (w/w) of each monofluorinated benzaldehyde, 180 mM pyruvate, 550 μL phosphate buffer (90 mM, pH 6.0, containing 1 mM $MgSO_4$, 10 mM KCl, 10% D_2O), sum spectrum of first five hours of reaction.

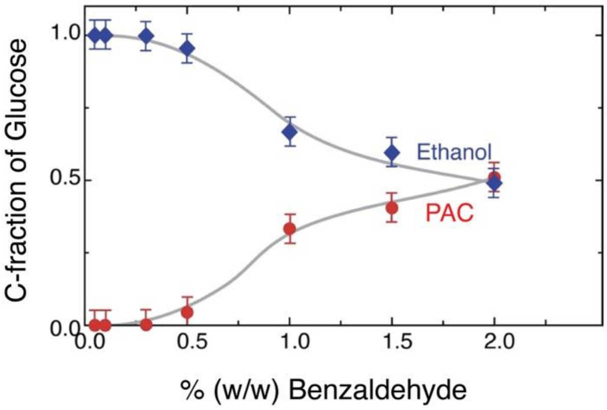

Figure S4. Internal competition of glucose-derived acetaldehyde for ethanol or PAC formation in dependence of weight-% benzaldehyde, showing massive redirecting of glucose carbon from alcoholic fermentation to PAC formation. Reaction conditions: variable weight-% benzaldehyde, 90 mM glucose in 550 μL phosphate buffer (90 mM, pH 6.0, containing 1 mM $MgSO_4$, 10 mM KCl, 10% D_2O), 100 mg commercial dry yeast, 303 K, 1 hour reaction time.

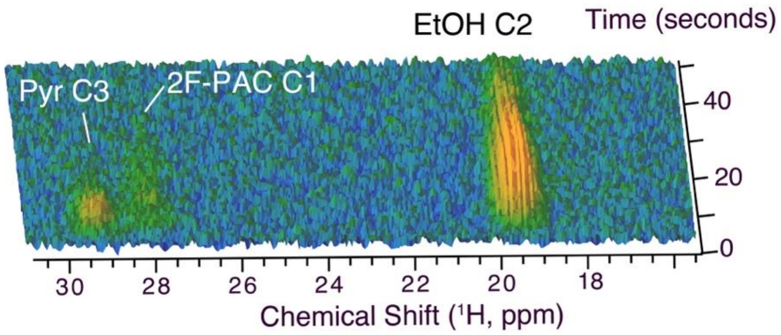

Figure S5. D-DNP NMR time series of ^{13}C NMR 1 D spectra acquired each 0.5 seconds, showing the influx of hyperpolarized D-[U-^{13}C,U-^{2}H]glucose into metabolites within 30 seconds in the presence of 0.5% (w/w) 2-fluorobenzaldehyde. 2F-PAC forms rapidly, as evidenced by the emergence of its methyl signal within few seconds.

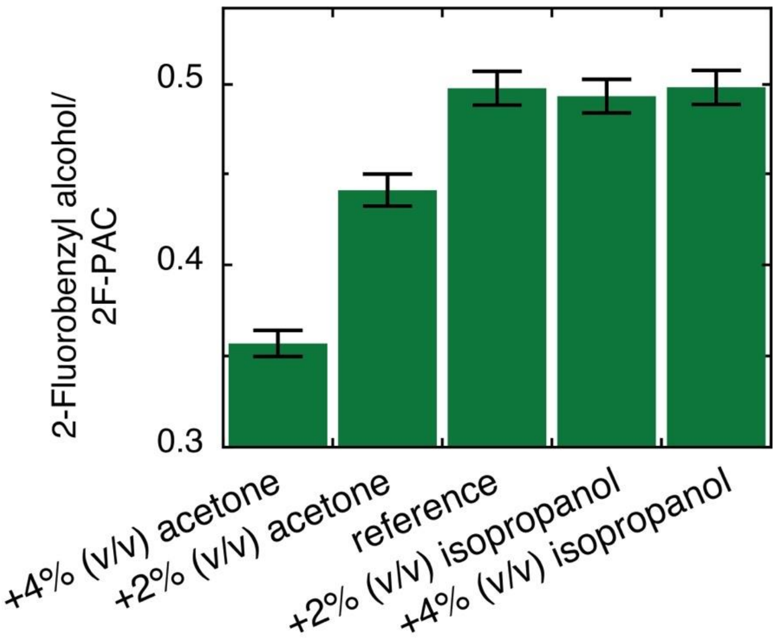

Figure S6. Effect of acetone and isopropanol on product distribution from 2-fluorobenzaldehyde in *S. cerevisiae*. Reaction conditions: 0.5 % (w/w) 2-fluorobenzaldehyde, 90 mM glucose, 550 mL phosphate buffer (90 mM, pH 6.0, containing 1 mM $MgSO_4$, 10 mM KCl, 10% D_2O), 100 mg commercial dry yeast, 303 K, 1 hour.

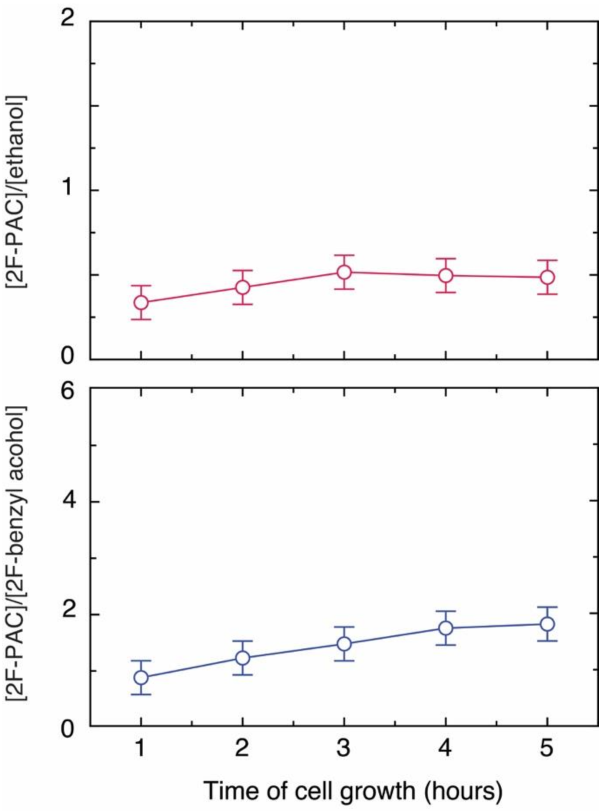

Figure S7. Effect of cell growth and uptake of oxidized reactant pyruvate on product distribution in the reaction between 2-fluorobenzaldehyde and glucose, akin to the reaction with pyruvate of main text Figure 9. The ratio between 2F-PAC and ethanol is plotted on the same scale as in Figure 9, while the ratio between 2F-PAC and 2-fluorobenzyl alcohol is plotted on a smaller scale than in Figure 9 due to significantly lower ratio. Reaction conditions: Preincubation of 100 mg commercial dry yeast in YPD for variable time, subsequent reaction of 0.5 % (w/w) 2-fluorobenzaldehyde, 90 mM glucose, identical number of cells in 550 μL phosphate buffer (90 mM, pH 6.0, containing 1 mM $MgSO_4$, 10 mM KCl, 10% D_2O), 303 K, 1 hour.

Table S1. Full ^{1}H and ^{13}C chemical shift assignments of benzaldehyde and its acyloin condensation product after whole cell catalysis with commercial yeast, as well as alcohol and acid in H_2O at 298 K.

Benzaldehyde	Carbon	δ^{1}H (ppm)	δ^{13}C (ppm)
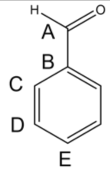	A	9.92	199.3
	B	-	138.2
	C	7.94	132.8
	D	7.62	131.9
	E	7.75	138.1

PAC	Carbon	δ^{1}H (ppm)	δ^{13}C (ppm)
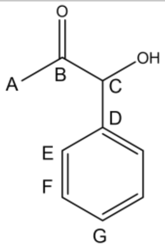	A	2.09	28.2
	B	-	214.1
	C	5.37	82.3
	D	-	139.8
	E	7.41	130.3
	F	7.62	131.8
	G	7.75	138.1

Benzyl alcohol	Carbon	δ^{1}H (ppm)	δ^{13}C (ppm)
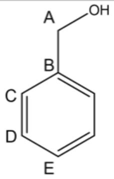	A	4.63	66.6
	B	-	142.9
	C	7.40	130.2
	D	7.36	130.5
	E	7.31	132.0

Benzoic acid	Carbon	δ^{1}H (ppm)	δ^{13}C (ppm)
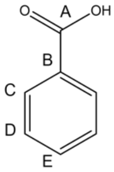	A	-	178.2
	B	-	133.8
	C	7.86	131.4
	D	7.47	130.9
	E	7.54	133.9

Table S2. Full ^{1}H and ^{13}C chemical shift assignments of 2-fluorobenzaldehyde and its acyloin condensation product after whole cell catalysis with commercial yeast, as well as alcohol and acid in H_2O at 298 K.

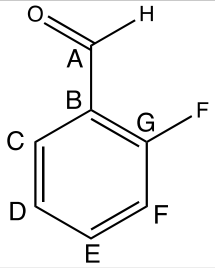

2-fluorobenzaldehyde	Carbon	$\delta^{1}H$ (ppm)]	$\delta^{13}C$ (ppm)
	A	10.18	194.4
	B	-	126.2
	C	7.87	132.6
	D	7.38	127.5
	E	7.74	140.0
	F	7.3	119.2
	G	-	166.7

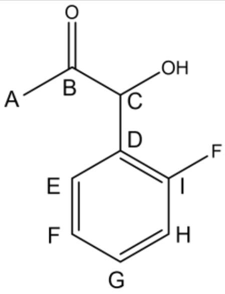

2F-PAC	Carbon	$\delta^{1}H$ (ppm)	$\delta^{13}C$ (ppm)
	A	2.14	28.1
	B	-	213.7
	C	5.54	77.0
	D	-	129.5
	E	7.39	132.5
	F	7.27	127.7
	G	7.46	134.0
	H	7.22	118.8
	I	-	162.9

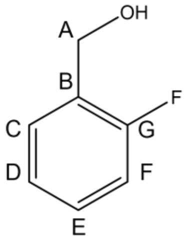

2-fluorobenzyl alcohol	Carbon	$\delta^{1}H$ (ppm)	$\delta^{13}C$ (ppm)
	A	4.69	60.7
	B	-	129.6
	C	7.43	132.8
	D	7.22	127.1
	E	7.39	132.6
	F	7.17	118.1
	G	-	163.3

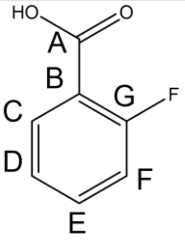

2-fluorobenzoic acid	Carbon	$\delta^{1}H$ (ppm)	$\delta^{13}C$ (ppm)
	A	-	171.4
	B	-	121.4
	C	7.89	134.4
	D	7.30	127.2
	E	7.64	138.0
	F	7.25	119.4
	G	-	164.1

Table S3. Full ^{1}H and ^{13}C chemical shift assignments of 3-fluorobenzaldehyde and its acyloin condensation product after whole cell catalysis with commercial yeast, as well as alcohol and acid in H_2O at 298 K.

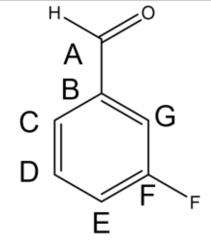

3-fluorobenzaldehyde	Carbon	δ^1H (ppm)	δ^{13}C (ppm)
	A	9.92	198.0
	B	-	140.5
	C	7.78	129.4
	D	7.63	133.9
	E	7.49	124.8
	F	-	165.5
	G	7.65	118.2

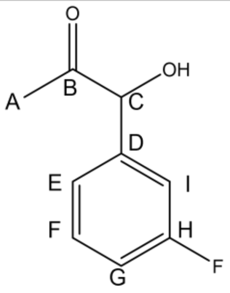

3F-PAC	Carbon	δ^1H (ppm)	δ^{13}C (ppm)
	A	2.11	28.1
	B	-	213.3
	C	5.37	81.6
	D	-	141.9
	E	7.23	126.1
	F	7.47	133.6
	G	7.17	118.4
	H	-	165.2
	I	7.16	116.8

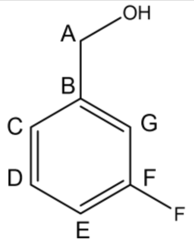

3-fluorobenzyl alcohol	Carbon	δ^1H (ppm)	δ^{13}C (ppm)
	A	4.63	65.9
	B	-	145.4
	C	7.19	125.3
	D	7.41	133.1
	E	7.08	117.0
	F	-	164.6
	G	7.13	116.4

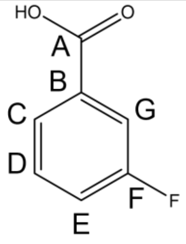

3-fluorobenzoic acid	Carbon	δ^1H (ppm)	δ^{13}C (ppm)
	A	-	172.4
	B	-	135.1
	C	7.82	128.1
	D	7.53	133.2
	E	7.40	123.1
	F	-	165.1
	G	7.71	118.9

Table S4. Full ^{1}H and ^{13}C chemical shift assignments of 4-fluorobenzaldehyde and its acyloin condensation product after whole cell catalysis with commercial yeast, as well as alcohol and acid in H_2O at 298 K.

4-fluorobenzaldehyde	Carbon	δ^{1}H (ppm)	δ^{13}C (ppm)
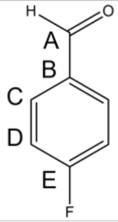	A	9.88	197.7
	B	-	135.1
	C	8.0	135.5
	D	7.33	119.2
	E	-	169.5

4F-PAC	Carbon	δ^{1}H (ppm)	δ^{13}C (ppm)
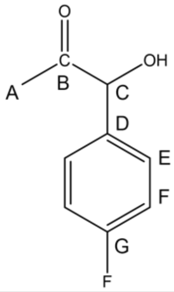	A	2.10	28.1
	B	-	214.1
	C	5.37	81.5
	D	-	135.6
	E	7.41	132.2
	F	7.19	118.5
	G	-	165.3

4-fluorobenzyl alcohol	Carbon	δ^{1}H (ppm)	δ^{13}C (ppm)
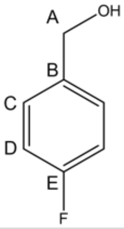	A	4.60	65.9
	B	-	138.6
	C	7.39	132.1
	D	7.13	117.9
	E	-	164.6

4-fluorobenzoic acid	Carbon	δ^{1}H (ppm) [ppm]	δ^{13}C (ppm)
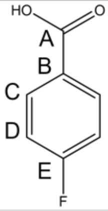	A	-	181.9
	B	-	129.1
	C	8.04	135.1
	D	7.23	118.4
	E	-	168.5
